# The SocioBox: A Novel Paradigm to Assess Complex Social Recognition in Male Mice

**DOI:** 10.3389/fnbeh.2016.00151

**Published:** 2016-08-11

**Authors:** Dilja Krueger-Burg, Daniela Winkler, Mišo Mitkovski, Fernanda Daher, Anja Ronnenberg, Oliver M. Schlüter, Ekrem Dere, Hannelore Ehrenreich

**Affiliations:** ^1^Max Planck Institute of Experimental MedicineGöttingen, Germany; ^2^DFG Research Center for Nanoscale Microscopy and Molecular Physiology of the BrainGöttingen, Germany; ^3^European Neuroscience InstituteGöttingen, Germany

**Keywords:** behavior, gender differences, mouse model, autism, schizophrenia, PSD-95

## Abstract

Impairments in social skills are central to mental disease, and developing tools for their assessment in mouse models is essential. Here we present the SocioBox, a new behavioral paradigm to measure social recognition. Using this paradigm, we show that male wildtype mice of different strains can readily identify an unfamiliar mouse among 5 newly acquainted animals. In contrast, female mice exhibit lower locomotor activity during social exploration in the SocioBox compared to males and do not seem to discriminate between acquainted and unfamiliar mice, likely reflecting inherent differences in gender-specific territorial tasks. In addition to a simple quantification of social interaction time of mice grounded on predefined spatial zones (zone-based method), we developed a set of unbiased, data-driven analysis tools based on heat map representations and characterized by greater sensitivity. First proof-of-principle that the SocioBox allows diagnosis of social recognition deficits is provided using male PSD-95 heterozygous knockout mice, a mouse model related to psychiatric pathophysiology.

## Introduction

A fundamental prerequisite for living in social communities is a highly complex set of social skills that governs interactions between individual members of a group. In consequence, impairments in these social skills, prominently prevalent in human psychiatric disorders such as autism and schizophrenia, have devastating consequences for individuals and society (Meyer-Lindenberg and Tost, 2012; Lai et al., 2014; Green et al., 2015). Major efforts are underway to understand molecular and cellular mechanisms that underlie social behaviors (Yizhar et al., 2011; Gunaydin et al., 2014; Hitti and Siegelbaum, 2014) and link disease-associated genetic constellations with social dysfunction (Tabuchi et al., 2007; Jamain et al., 2008; Schmeisser et al., 2012; Tantra et al., 2014). This interest is reflected in a 10-fold increase in the number of publications on mouse models of psychiatric diseases over the past 15 years.

This research has been hampered by the limited availability of reliable tools assessing complex social functions. Like humans, mice are capable of a rich set of social behaviors (Singleton and Krebs, 2007), but virtually all current tests assess only simple two-way discriminations between a mouse and an inanimate object or between a familiar and an unfamiliar mouse (Moy et al., 2004; Silverman et al., 2010; Kas et al., 2014). Accordingly, they are likely to underestimate the social competencies of mice, obscuring subtle alterations in disease-related social functions. Here we present a novel paradigm for standardized measurement of more complex social recognition in mice, proving for the first time that male mice are easily capable of distinguishing between 5 mice. This paradigm provides a valuable tool for studying the circuitry underlying social recognition in wildtype mice as well as for identifying mechanisms by which disease-associated mutations may result in impaired social skills.

## Materials and Methods

### Animals

Wildtype (WT) mice were purchased from Janvier (C57BL/6JRj) or Charles River (C3H/HeNCrl and BALB/cAnNCrl). The age of the mice upon arrival ranged from 3 weeks (males) to 6 weeks (females). Mice were group-housed in standard cages (36.5 cm × 20.7 cm × 14 cm, 4–5 mice per cage of the same gender and strain), in rooms separated by gender (to avoid olfactory contact). PSD-95 heterozygous knockout mice (Yao et al., 2004) on C57BL/6J background were obtained from the laboratory of Oliver Schlüter and bred in the animal facility at the Max Planck Institute of Experimental Medicine. An experimental cohort of male PSD-95 heterozygous knockout mice (PSD-95^+/-^) and male WT littermate controls was generated from PSD-95 heterozygous breeding pairs and was housed in large cages (60 cm × 38 cm × 20 cm) in groups of 17 mice of mixed genotypes. Food and water were provided *ad libitum*. The housing room was maintained on a 12 h light–dark cycle (lights off at 7 pm) at 20–22°C. All experiments were approved by the local Animal Care and Use Committee in accordance with the German Animal Protection Law.

### Experimental Setup

Experiments on WT mice were conducted using groups of 10 mice (‘experimental mice’) of the same gender and strain (C57Bl/6J, C3H or BALB/c) as specified, aged 12–15 weeks at the beginning of testing. C3H mice were used as interaction partners (‘stimulus mice’) for all experiments, based on prior reports that this strain shows robust social interaction in a test situation (Moy et al., 2007). Stimulus mice were gender-matched with the experimental mice and were aged 12–15 weeks at the beginning of testing. All experiments were conducted during the light phase of the day (from 9 am to 6 pm) at a light intensity of 10–15lux.

Experiments on PSD-95 heterozygous knockout mice were conducted using a group of 14 PSD-95^+/+^ mice and 20 PSD-95^+/-^ mice, aged 16 weeks at the beginning of testing. C3H mice were again used as stimulus mice, and testing was performed during the light phase of the day (from 9 am to 6 pm) by an investigator blind to genotype. The experiment described here was part of a test battery conducted on this cohort of mice, the complete results of which will be reported elsewhere.

### SocioBox Apparatus

**FIGURE 1:**
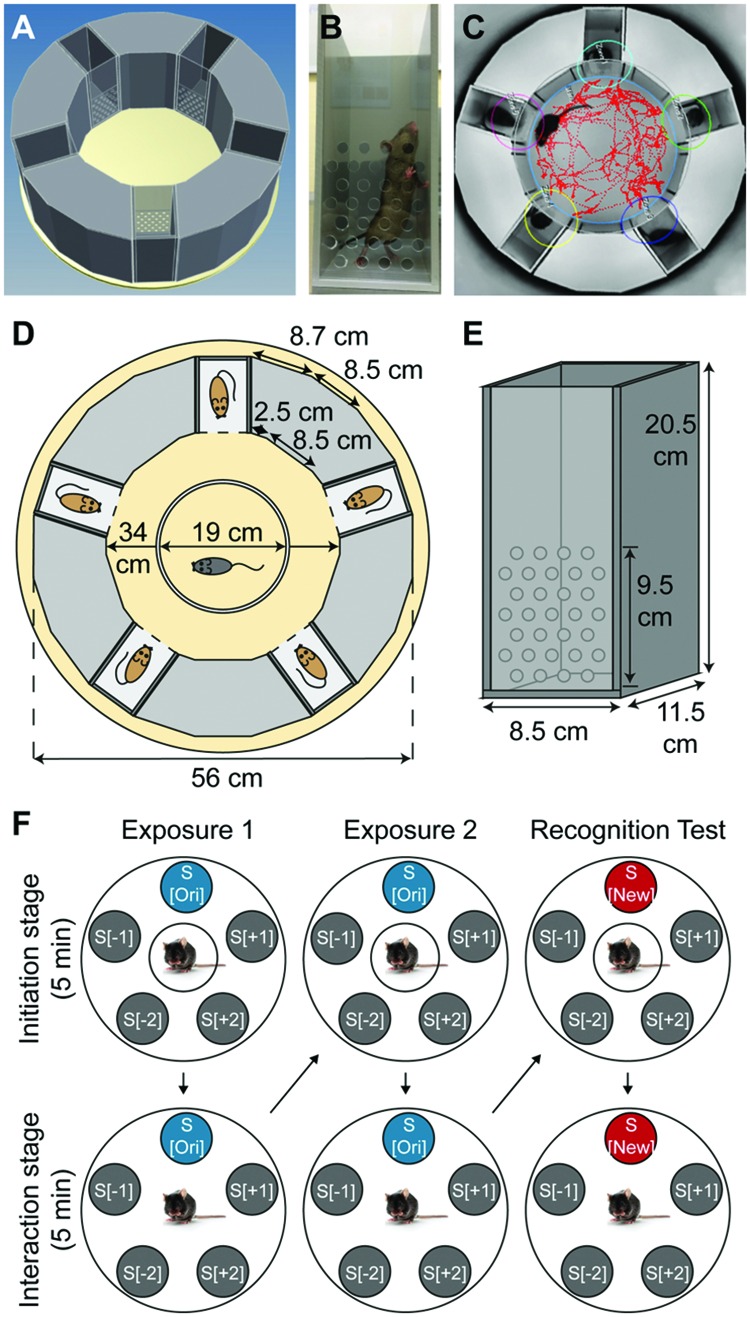
**Design of the SocioBox paradigm. (A)** 3-dimensional representation of the SocioBox apparatus, showing the ring of stimulus mouse inserts around the central arena containing the experimental mouse. **(B)** Photograph of a stimulus mouse insert. **(C)** Image from the Viewer3 software, showing the digital mouse track and interaction zones overlaid on a photograph from the overhead camera. **(D)** Construction diagram of the SocioBox chamber. The floor plate is shown in light yellow; the 5 inserts are shown in dark gray (each containing a stimulus mouse); the plastic walls are shown in light gray; the circular partition is shown in white. **(E)** Construction diagram of an insert. The insert walls are shown in dark gray; the detachable clear front slider is shown in light gray. The dimensions of each element are indicated with arrows. **(F)** Flow chart of the test session on day 4. The large circles represent the SocioBox apparatus; the small filled circles represent the stimulus mouse inserts; the circles around the experimental mouse in the center represent the central circular partition. The columns represent the 3 phases of the test session, exposure 1, exposure 2, and recognition test. The rows represent the 2 stages, the initiation stage (experimental mouse separated from the stimulus mice by the circular partition) and the interaction stage (circular partition removed). Arrows indicate the progression of the experiment. S[ori] = original stimulus mouse; S[new] = new stimulus mouse; S[-1] and S[+1] = stimulus mice immediately adjacent to the S[ori]/S[new] mouse; S[-2] and S[+2] = stimulus mice opposite the S[ori]/S[new] mouse.

The SocioBox apparatus (**Figures 1A–E**; **Table 1**) was constructed by the machine shop at the Max Planck Institute of Experimental Medicine. It consisted of a plastic ground plate, an outer ring of five rectangular removable boxes (‘inserts’) separated by fixed dividers of gray plastic, and a central open arena (diameter of the outer ring = 56 cm, diameter of the central arena = 34 cm, height of the inserts and the dividers = 20.5 cm). Experimental mice were placed in the central arena, while stimulus mice were placed in the inserts in the outer ring. The inserts (width 8.5 cm, length 11.5 cm, height 20.5 cm) consisted of 3 walls of gray plastic and 1 front slider of clear plastic, facing the center of the apparatus, with 31 holes (diameter 0.8 cm) to permit social interaction and exchange of odors between the experimental mice and the stimulus mice (**Figures 1B,E**). The front slider was removable to facilitate cleaning of the inserts between mice. Within the central arena, a circular partition of white plastic (diameter 19 cm, height 18 cm, without a floor) served to spatially and visually separate the experimental mice from the inserts containing the stimulus mice during the initiation stage of the test paradigm (see below).

**Table 1:** Glossary of the SocioBox terminology.

Category	Term	Explanation
**Apparatus**	Central arena	Open area in the center of the SocioBox that contains the experimental mouse
	Insert	Removable boxes that contain the stimulus mice
	Partition	Opaque removable divider that separates the experimental and stimulus mice during initiation
	Interaction zone	Area defined in the Viewer3 software that is adjacent to each insert and used to calculate interaction time
**Mice**	Experimental mouse	Mouse being tested for social recognition
	Stimulus mouse	Interaction partner for experimental mice
	Original stimulus mouse (S[ori] mouse)	Stimulus mouse that is present in exposures 1 and 2, but that is removed for the recognition test
	New stimulus mouse (S[new] mouse)	Stimulus mouse that is inserted instead of the S[ori] mouse during the recognition test
	Constant stimulus mouse (S[con] mouse)	Stimulus mice that are present in all 3 phases of the test session
	Unfamiliar stimulus mouse	Stimulus mouse that has never been seen by the experimental mouse
	Newly acquainted stimulus mouse	Stimulus mouse that was seen in a previous phase of the test session, but not prior to the current day
**Paradigm**	Test session	Day 4 of the paradigm: Test session lasting approx. 40 min, consisting of 3 test phases
	Test phase	Each test phase (exposure 1, exposure 2, and recognition test) lasts 10 min and consists of 2 min × 5 min stages, the initiation and interval stages
	Exposures 1 and 2	First and second phase of the test session, in which the experimental mouse encounters 1 S[ori] mouse and 4 S[con] mice
	Recognition test	Third phase of the test session, in which the S[ori] mouse is replaced by the S[new] mouse
	Initiation stage	First 5 min of each test phase, in which the experimental and stimulus mice are separated visually and spatially by the opaque partition
	Interaction stage	Second 5 min of each test phase, in which social interaction can occur between experimental and stimulus mice
**Data analysis**	Social Recognition Index (SRI)	= (Interaction time with S[new] mouse) – (Mean interaction time with S[con] mice)
	Spatial heat map	Analysis showing mean localization of all mice per group in a given test phase
	Temporal distance heat map	Analysis showing the distance of each experimental mouse from the point of origin (i.e., the center for exposures 1 and 2, S[new] for the recognition test) across the 5 min of the test phase

### Video Tracking

An overhead video camera (Samsung) was mounted approximately 130 cm above the SocioBox apparatus. This camera was connected to a computer located in a separate room, enabling the experimenter to record the test session without being present in the room during testing. Videos were acquired with a temporal resolution of 25 frames per sec. An automated software (Viewer3, Biobserve) was used to track the experimental mouse and calculate the time spent in interaction with each stimulus mouse (defined as time in which the experimental mouse was located within a predefined interaction zone adjacent to the insert, see **Figure 1C**).

### Habituation of Experimental and Stimulus Mice

Prior to the test session, experimental and stimulus mice were habituated separately to the SocioBox apparatus for 3 consecutive days. This extensive habituation paradigm served to reduce anxiety in the mice, which is known to result from exposure to a novel open environment (Calhoon and Tye, 2015) and which may substantially interfere with social interest and recognition.

Stimulus mice were habituated to the inserts within the apparatus, but in the absence of an experimental mouse. Each mouse received 3 habituation sessions per day, each lasting for 10 min. For this purpose, the mouse was placed in the insert, and the insert was carefully placed in its slot in the outer ring. For the first 5 min, the circular partition in the central arena was present, and it was then removed for the last 5 min to mimic the test situation. The inserts were cleaned after every use, first with running tap water to remove urine and feces, then with 70% ethanol, then again with tap water to remove the smell of the ethanol. The inserts were then dried with paper tissue before the next use.

Experimental mice were habituated to the central arena with the inserts in place, but in the absence of any stimulus mice. Each mouse received 1 habituation session per day, lasting for 10 min. For this purpose, the mouse was placed in the arena inside the circular partition for 5 min. After this time, the partition was removed to mimic the test situation, and the mouse was allowed to explore the entire arena. The arena was cleaned after every use, first with wet tissues, then with 70% ethanol, then again with wet tissues to remove the smell of the ethanol. Both the floor of the arena (including the space underneath the inserts, which were lifted briefly for cleaning purposes) and any parts of the walls that had been in direct contact with the experimental mouse (including the front sliders of the inserts) were cleaned in this manner.

### Test Session

On the day of the test session (day 4 of the paradigm, after 3 habituation days), mice were exposed to the light conditions (10–15lux) of the experimental procedure in a separate room for approximately 30 min before testing. At the beginning of each test session, 6 assigned C3H stimulus mice were placed into 6 inserts and they stayed in these inserts throughout the test session. Five inserts were then transferred to the room with the SocioBox apparatus and were placed inside the apparatus. The 6th insert stayed in the separate room at the aforementioned light conditions until it was introduced to the apparatus during the recognition test phase. This procedure ensured that all stimulus mice spent equal time inside the inserts, since the activity of the stimulus mice within the insert decreased over time.

Each test session consisted of 3 phases, exposure 1, exposure 2, and recognition test (**Figure 1F**). To begin the test session, the experimental mouse was placed into the central arena inside the white Plexiglass circular partition, which separated the experimental mouse from the stimulus mice spatially and visually. After 5 min of recovery (‘initiation stage’), the circular partition was lifted, and the experimental mouse was allowed to freely explore the stimulus mice in their inserts for 5 min (‘interaction stage’). At the end of exposure 1, the experimental mouse was removed from the apparatus and placed in the cage used to transport the mouse into the testing room. The central arena was cleaned as described for the habituation session, and the experimental mouse was then returned to the central arena inside the circular partition. Exposure 2 followed immediately and consisted of the same initiation stage and interaction stage. At the end of exposure 2, the experimental mouse was again removed from the apparatus and the central arena was cleaned as above. At this point, 1 of the stimulus mice (the ‘original stimulus mouse’, S[ori]) was removed with its insert, and the insert containing the 6th stimulus mouse (the ‘new stimulus mouse’, S[new]) was introduced in its place. The other stimulus mice (‘constant stimulus mice’, S[-2], S[-1], S[+1], and S[+2]) remained in the same position throughout the testing paradigm to avoid potential interference of object location memory (Dere et al., 2005; Murai et al., 2007). After this exchange was completed, the experimental mouse was returned to the circular partition and the recognition test was conducted, again consisting of an initiation stage and an interaction stage. At the end of the test session, the experimental mouse and the 6 stimulus mice were returned to their home cages, and the central arena and inserts were cleaned as described for the habituation phase.

In order to avoid fatigue and social disinterest of the stimulus mice due to repeated exposure to multiple experimental mice, several sets of stimulus mice were used for each experiment, and no stimulus mouse was used for >3 test sessions per day. Moreover, to eliminate any spatial biases on the part of the experimental mouse, the position of the S[ori]/S[new] stimulus mouse within the SocioBox chamber was rotated for each new experimental mouse. Regardless of the position of the S[ori]/S[new] stimulus mouse, the stimulus mouse immediately clockwise was designated as S[+1] for data alignment purposes, the next stimulus mouse clockwise was S[+2], the stimulus mouse immediately anti-clockwise was S[-1], and the next stimulus mouse anti-clockwise was S[-2]. For each experimental mouse, the spatial location of each stimulus mouse was the same across exposure 1, exposure 2, and recognition test (except for the S[ori]/S[new] exchange).

### Analysis of Data Generated by the Viewer3 Software

The amount of time spent by the experimental mouse in each of the predefined zones during the 5 min of the interaction stage of each test was automatically calculated by the Viewer3 software. These data were aligned to the position of the S[ori]/S[new] stimulus mouse for purposes of statistical analysis and graphical representation.

### Data-Driven Analysis Using Spatial Heat Map Visualization

2-dimensional, spatial heat maps depicting average animal location were generated with the open source FIJI image processing package (Schindelin et al., 2012), using the C57BL/6J male data set as an example (**Figures 4A–K**). A custom-written FIJI macro imported and then plotted a 1-pixel-diameter point of intensity 1 at each of the 7500 body coordinates originally generated by the Viewer3 software. This step produced an image stack consisting of 7500 frames for each of the animals. The frames of this stack were summed to generate 1 superimposed image containing all plotted coordinates an individual mouse had occupied during the 5 min of the interaction stage for each test (**Figure 4A**). Geometrical rotations were applied to the individual animal data sets so that the S[ori]/S[new] stimulus mouse was always aligned in the top position. The 2-dimensional heat maps depicting an animal group were then made by adding up the images of the summed coordinates of the individual animals belonging to that group (**Figure 4B**). Gaussian-weighted noise reduction filtering was applied with a 5-pixel radius that revealed 5 average locations the animals preferred during the testing period (**Figure 4C**). The image contrast was enhanced by applying a polychromatic lookup table to more easily recognize potential hotspots signified by aggregations of pixels with similar values. In a next step, thresholding was applied that segmented out 5 regions preferred by the animals (**Figure 4D**). The resulting binary images were used to generate a mask containing 5 regions of interests (ROIs) for each phase of the experiment (**Figure 4E**). Finally, ROIs were applied to the original, non-filtered, summed images of each animal to quantify the number of pixels per individual ROI, where 25 pixels correspond to 1 sec (**Figure 4F**). For the analysis of the PSD-95^+/-^ experiment (**Figures 6A–D**), heat map representations were first generated separately for each genotype and then summed to generate a combined binary image and mask for quantification.

### Data-Driven Analysis Using Temporal Heat Map Visualization

To obtain a single, global visual representation of mouse behavior during each test phase, heat maps were generated depicting the distance of the experimental mouse from the center of the arena (exposure 1) or the S[new] stimulus mouse (recognition test) across the 5 min of the recording, using the male and female BALB/c data set as an example (**Figures 5A–D**). First, the body coordinates generated by the Viewer3 tracking of each animal were imported into the Calc spreadsheet software of the open source LibreOffice5 (libreoffice.org) software package. Geometrical rotations were applied to the individual animal data sets so that the S[ori]/S[new] stimulus mouse was always aligned in the top position. Next, the distance of the experimental mouse to the point of origin (defined as the center of the arena for exposure 1 and the S[new] stimulus mouse for the recognition test, see **Figures 5A,B**) was calculated for each of the recorded 7500 timepoints (25 frames per sec × 300 sec of recording). The resulting distances were imported into FIJI in order to generate a multi-column image in which each column consists of 7500 distance recordings of an individual animal organized in a temporal manner (**Figures 5C,D**). In order to quantify these data, FIJI was used to generate histograms showing the frequencies with which individual distances occur (**Figures 5E,F**). For the recognition test (**Figures 5D,F**), 3 distance frequency populations emerged which corresponded to (i) the location of the new stimulus mouse S[new], (ii) the location of the constant stimulus mice immediately adjacent to the new stimulus mouse, i.e., S[-1] and S[+1], and (ii) the location of the constant stimulus mice opposite to the new stimulus mouse, i.e., S[-2] and S[+2]. To better visualize these 3 populations, a custom green–blue–red lookup table was created, where the colors correspond to the distances 0–8, 10–24, and 26–34 cm, respectively.

### Statistical Analysis

**FIGURE 2:**
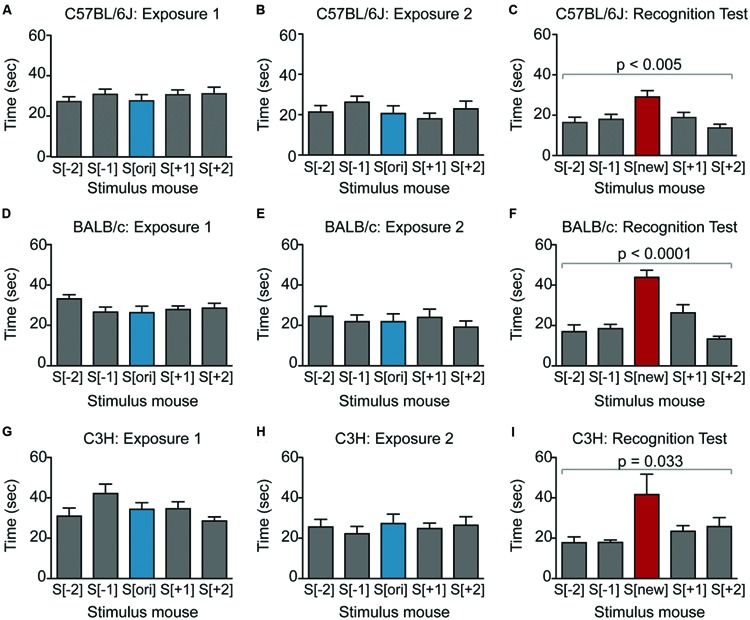
**Male mice from multiple strains show robust social recognition for 5 stimulus mice in the SocioBox paradigm. (A–C)** Interaction time of male C57BL/6J mice with each stimulus mouse during **(A)** exposure 1, **(B)** exposure 2, and **(C)** recognition test. **(D–F)** Interaction time of male BALB/c mice with each stimulus mouse during **(D)** exposure 1, **(E)** exposure 2, and **(F)** recognition test. **(G–I)** Interaction time of male C3H mice with each stimulus mouse during **(G)** exposure 1, **(H)** exposure 2, and **(I)** recognition test. Statistical analysis was conducted using one-way ANOVA, summarized in **Table 2**. Data are expressed as interaction time in sec (mean ± SEM).

**FIGURE 3:**
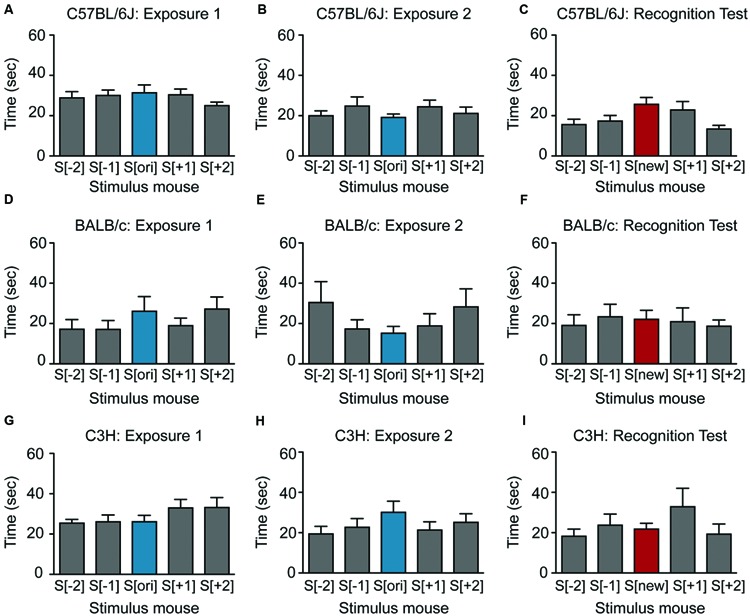
**Female mice show little or no social recognition in the SocioBox paradigm. (A–C)** Interaction time of female C57BL/6J mice with each stimulus mouse during **(A)** exposure 1, **(B)** exposure 2, and **(C)** recognition test. **(D–F)** Interaction time of female BALB/c mice with each stimulus mouse during **(D)** exposure 1, **(E)** exposure 2, and **(F)** recognition test. **(G–I)** Interaction time of female C3H mice with each stimulus mouse during **(G)** exposure 1, **(H)** exposure 2, and **(I)** the recognition test. Statistical analysis was conducted using one-way ANOVA, summarized in **Table 2**. Data are expressed as interaction time in sec (mean ± SEM).

**FIGURE 4:**
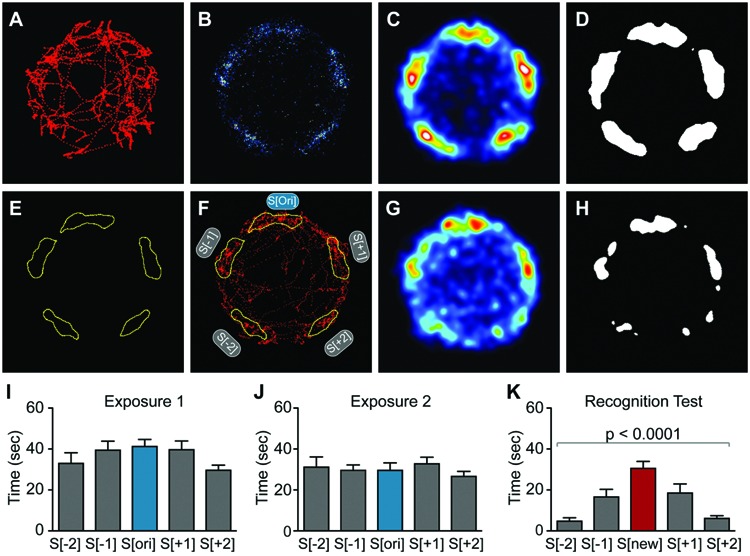
**Spatial heat map analysis improves sensitivity of detection in the SocioBox paradigm. (A–C)** Generation of the spatial heat map, using data for C57BL/6J males (exposure 1, corresponds to **Figure 2A**) as an example: **(A)** Example of body coordinates of a single mouse, exported from the Viewer3 software into FIJI. **(B)** Overlay of tracks from all C57BL/6J male mice for exposure 1. **(C)** Gaussian-weighted noise reduction filtering of the overlaid tracks to generate a spatial heat map. **(D)** Binary image created by application of a threshold to the spatial heat map. **(E)** Example of ROIs created from a binary image. **(F)** Example of the application of the mask created from the ROIs to the track of a single mouse. **(G,H)** Spatial heat map of data from the C57BL/6J male recognition test (corresponds to **Figure 2C**). **(G)** Heat map showing the combined location of the experimental mice during the recognition test, and **(H)** thresholded image. **(I–K)** Quantification of the time spent by male C57BL/6J mice in the 5 ROIs during **(I)** exposure 1, **(J)** exposure 2, and **(K)** recognition test (compare to **Figures 2A–C**). Statistical analysis was conducted using one-way ANOVA, summarized in **Table 2**. Data are expressed as interaction time in sec (mean ± SEM).

**FIGURE 5:**
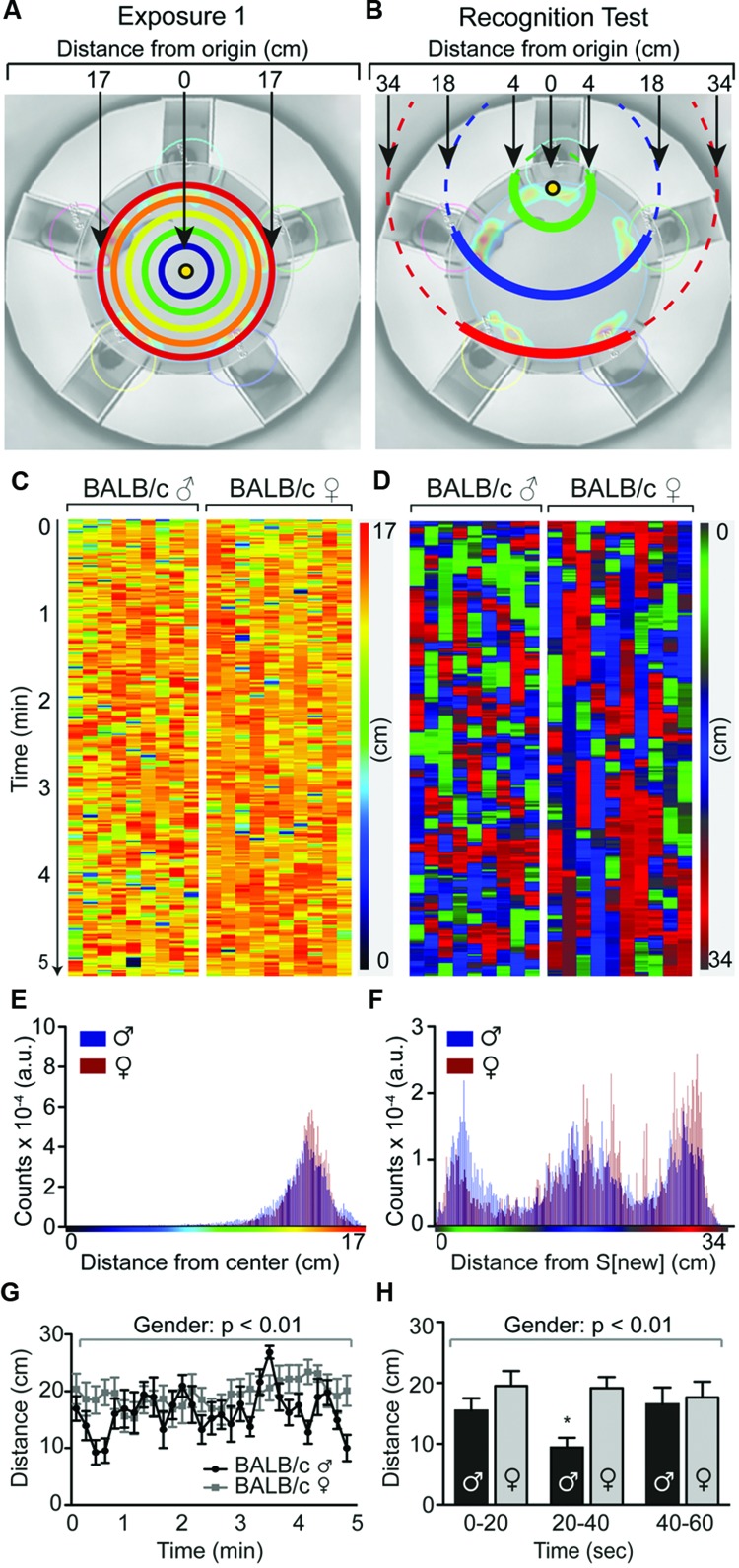
**Temporal distance heat maps identifies timing of social interaction in the SocioBox paradigm.** Temporal heat map of the recognition test in male and female BALB/c mice, showing the distance of individual mice from the new stimulus mouse over time. **(A,B)** Schematic diagram showing the method used to calculate the distance of the experimental mouse from the point of origin, i.e., **(A)** the center of the arena during exposure 1 (or exposure 2, not shown); or **(B)** the new stimulus mouse during the recognition test. **(C,D)** Temporal distance heat maps showing the distance of individual BALB/c mice from the point of origin at each time point during **(C)** exposure 1 and **(D)** the recognition test. Each experimental mouse is represented in 1 column, with the vertical location corresponding to time, and color and intensity corresponding to the distance from the new stimulus mouse (as indicated in the calibration bar). **(E,F)** Frequency distributions of distances found in male (blue) and female (red) animals within the temporal distance heat map of **(E)** exposure 1 and **(F)** the recognition test. In the recognition test, 3 distance populations emerge, which correspond to the location of the new stimulus mouse S[new], the location of the constant stimulus mice immediately adjacent to the new stimulus mouse, i.e., S[-1] and S[+1], and the location of the constant stimulus mice opposite to the new stimulus mouse, i.e., S[-2] and S[+2]. Based on this distribution, the color intensity and shade in the temporal heat map for the recognition test were assigned as follows: Green = distance 0–8 cm, blue = distance 10–24 cm, red = distance 26–34 cm. **(G,H)** Quantification of the average distance of male and female experimental mice from the new stimulus mouse **(G)** across the 5 min of the recognition test and **(H)** during the first minute of the recognition test, analyzed in 20 sec time bins. Statistical analysis was conducted using a repeated measures two-way ANOVA with gender as between-subjects factor and time as within-subjects factor. Data are expressed as distance traveled in cm (mean ± SEM).

**FIGURE 6:**
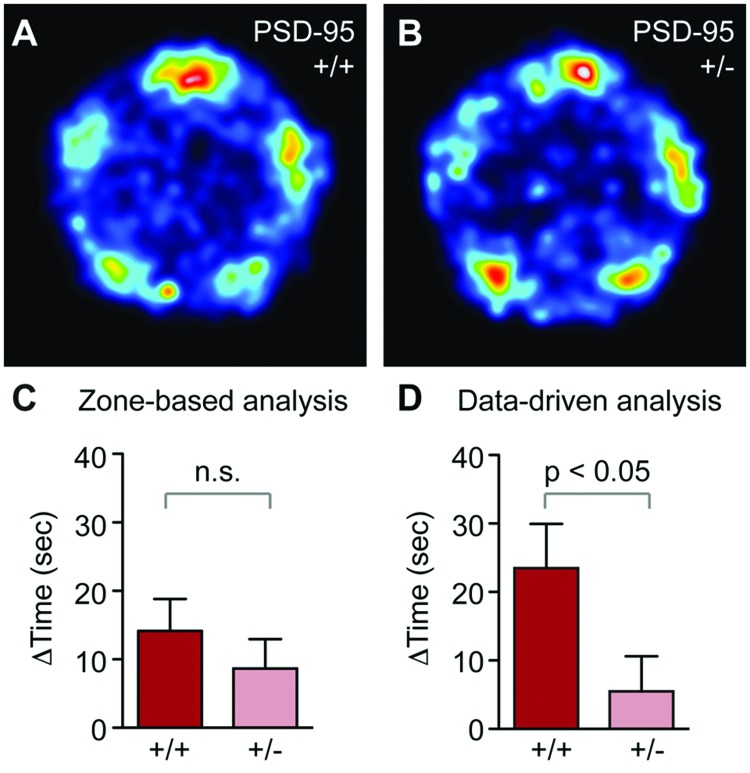
**PSD-95^+/-^ mice show reduced social recognition in the SocioBox paradigm. (A,B)** Heat map showing the localization of **(A)** PSD-95^+/+^ and **(B)** PSD-95^+/-^ mice during the recognition test. **(C,D)** Comparison of the SRI obtained for PSD-95^+/+^ and PSD-95^+/-^ mice using the zone-based **(C)** and data-driven **(D)** analysis methods. Statistical analysis was conducted using an unpaired two-tailed Student’s *t*-test. Data are expressed as time difference in sec (mean ± SEM).

Statistical analysis was performed using the Prism software v5.0c (GraphPad). Data were first analyzed for outliers using the Grubbs test outlier calculator (based on the method of Grubbs (1969) and calculated on the GraphPad website, http://graphpad.com/quickcalcs/Grubbs1.cfm, with a significance level of alpha = 0.01). The following parameters were considered for detecting outliers: Interaction time with each of the 5 stimulus mice (**Figures 2A–I**, **3A–I**, and **4J,K**) or social recognition index (SRI) (**Figures 6C,D**). No outlier analysis was conducted for **Figures 5G,H** due to the large number of data points involved. Mice for which an outlier was identified in a given data set were excluded from further analysis for this data set. This affected the following data sets: BALB/c males, exposures 1 and 2 (**Figures 2D,E**, 1 mouse); C57BL/6J females, recognition test (**Figure 3C**, 1 mouse); BALB/c females, exposure 2 (**Figure 3E**, 3 mice); PSD-95 experiment, PSD-95^+/+^ mice (**Figure 6D**, 1 mouse), PSD-95^+/-^ mice (**Figure 6D**, 2 mice). Moreover, 1 mouse was excluded entirely from the male C57BL/6J data set due to technical issues (escape of a stimulus mouse during the recognition test). Data sets were then analyzed using repeated measures one-way ANOVA (**Figures 2–4**), repeated measures two-way ANOVA with gender as between-subjects factor and time as within-subjects factor (**Figure 5**), or two-tailed unpaired Student’s *t*-test (**Figure 6**).

## Results and Discussion

After a series of optimization experiments (see Supplement for details), we developed a circular behavioral chamber (‘SocioBox’, see **Table 1** for nomenclature), in which the mouse of interest (‘experimental mouse’) was placed in a central arena surrounded by a ring of 5 unfamiliar mice (‘stimulus mice’) (**Figures 1A–D**). The stimulus mice were held in rectangular inserts facing the center of the chamber, with holes in the clear front panel to permit exchange of odors (**Figures 1B,E**). The movement of the experimental mouse was recorded by an overhead camera (**Figure 1C**), and the interaction time with each stimulus mouse (i.e., the time spent within a predefined zone in front of each insert, see **Figure 1C**), was calculated using an automated software (Viewer3, Biobserve).

Using this experimental setup, we established a testing paradigm that produces robust social recognition in wildtype mice (see Materials and Methods for details). Briefly, mice were first individually habituated to the apparatus for 3 consecutive days to eliminate any novelty-related confounds. On day 4, a test-session was conducted, consisting of 3 consecutive phases: Exposure 1, exposure 2, and recognition test (**Figure 1F**). In each phase, the experimental mouse was first placed into the central arena inside an opaque circular partition, which separated the experimental mouse from the 5 stimulus mice spatially and visually. After 5 min of exploration (‘initiation stage’), the partition was lifted and the experimental mouse was allowed to freely explore the stimulus mice in their inserts for 5 min (‘interaction stage’). For each experimental mouse, the identity and spatial position of the 5 stimulus mice was equal during exposures 1 and 2, while for the recognition test, 1 of the 5 stimulus mice (the ‘original stimulus mouse’, S[ori]) was replaced with an unfamiliar stimulus mouse (the ‘new stimulus mouse’, S[new]). The other stimulus mice (‘constant stimulus mice’, S[-2], S[-1], S[+1] and S[+2]) remained in their inserts in the same position throughout the testing paradigm. To control for spatial bias effects, the position of the S[ori]/S[new] stimulus mouse within the SocioBox chamber was rotated for each new experimental mouse. At the end of each phase, the experimental mouse was removed briefly while the arena was cleaned, and then returned to the circular partition to begin the next phase.

To validate our new paradigm, we first assessed social recognition in male C57BL/6J mice, commonly used as genetic background strain in animal models of psychiatric disorders (**Figures 2A–C**; **Table 2**). During exposure 1, experimental mice displayed substantial interest in the 5 stimulus mice (**Figure 2A**; **Table 2**), which decreased slightly during exposure 2 (**Figure 2B**; **Table 2**), likely due to habituation to these stimulus mice. During the recognition test, experimental mice spent even less time with the constant stimulus mice, but interacted significantly more with the new stimulus mouse (**Figure 2C**; **Table 2**). Based on the well-documented tendency of rodents to interact more with a novel object or individual than with a familiar one (Bevins and Besheer, 2006; Macbeth et al., 2009), this difference in interaction time likely reflected the ability of the experimental mouse to distinguish between the previously encountered stimulus mice and the new stimulus mouse. To provide a quantitative parameter of social recognition that would facilitate direct comparison between groups of mice, we generated a single SRI for each experimental mouse, calculated as the difference in time spent with the new stimulus mouse S[new] and with the average of the 4 constant stimulus mice (**Table 2**). Together, our data indicate that C57BL/6J male mice can distinguish an unfamiliar mouse from at least 4 other newly acquainted mice, and that the paradigm presented here enables robust assessment of this social recognition.

**Table 2:** Statistical analysis of data presented in **Figures 2–4**.

			Exposure 1 One-way ANOVA			Exposure 2 One-way ANOVA			Recognition Test One-way ANOVA			Recognition TestSRI^1^		
Gender	Strain	Figure	*p*-value	*F*-value	DF (n;d)	*p*-value	*F*-value	DF (n;d)	*p*-value	*F*-value	DF (n;d)	Ave ± SEM
Male	C57BL/6J	2A–C	0.759	0.47	4;32	0.466	1.57	4;32	0.002	5.64	4;32	12.3^2^ ± 3.9
	BALB/c	2D–F	0.261	1.38	4;36	0.899	0.26	4;32	<0.0001	17.07	4;32	25.1^2,3^ ± 3.1
	C3H	2G–I	0.138	1.87	4;36	0.895	0.27	4;36	0.033	2.96	4;36	20.4 ± 11.3
	C57BL/6J^4^	4I–K	0.208	1.17	4;32	0.792	0.42	4;32	<0.0001	12.05	4;32	19.0 ± 3.1
Female	C57BL/6J	3A–C	0.646	0.63	4;36	0.673	0.59	4;36	0.078	2.33	4;32	8.4 ± 4.3
	BALB/c	3D–F	0.568	0.74	4;36	0.525	0.82	4;24	0.963	0.15	4;36	1.6^3^ ± 3.2
	C3H	3G–I	0.329	1.20	4;36	0.433	0.98	4;36	0.435	0.97	4;36	-1.7 ± 2.5

*^1^SRI, Social recognition index, calculated as (Interaction time with S[new]) – (Mean interaction time with S[con]). ^2^Comparison of SRI for C57BL/6J vs. BALB/c males: *p* < 0.05, Student’s unpaired two-tailed *t*-test. ^3^Comparison of SRI for BALB/c males vs. females: *p* < 0.0001, Student’s unpaired two-tailed *t*-test. ^4^Re-analysis of C57BL/6J male data using data-driven analysis method (**Figure 4**)*.

To confirm that our results were not confounded by olfactory cues left by the experimental mouse on the front sliders of the inserts via touching or nose poking, we repeated the experiment with a slightly modified protocol, which included a complete exchange of sliders for freshly cleaned ones during the recognition test. This procedure did not substantially affect performance in the recognition test (SRI, *p* = 0.02), and since it extended the length of the procedure and somewhat distracted the mice, we did not include the slider exchange in our subsequent experiments.

We next asked whether our paradigm would be similarly suitable for other mouse strains. Male BALB/c and C3H mice also displayed substantial social interest in exposures 1 and 2 and significant preference for the new stimulus mouse in the recognition test (**Figures 2D–I**; **Table 2**), confirming the validity of our paradigm. Interestingly, the SRI for BALB/c mice was significantly higher than for C57BL/6J mice (25.1 ± 3.1 vs. 12.3 ± 3.9, respectively), indicating that male BALB/c mice show particularly pronounced social recognition skills under our conditions. In striking contrast, female mice of all 3 strains exhibited markedly lower preference for the new stimulus mouse (**Figures 3A–I**; **Table 2**), uncovering an essential gender difference in this behavior.

All of the above data originated from the Viewer3 software, which uses predefined spatial zones to quantify social interaction time (**Figure 1C**). An important limitation of this zone-based method is that it requires prior assumptions regarding the behavior of experimental mice, and that it may either over- or underestimate the time spent in social interaction if the predefined zones do not accurately match movement patterns of mice. To overcome this limitation, we developed a new set of unbiased, data-driven analysis tools based on heat map representations.

First, we generated spatial heat maps showing the average localization of all mice in a given test session, using the male C57BL/6J data set as example (**Figures 4A–H**). We identified 5 clearly preferred spatial locations in exposure 1 that corresponded to the location of the 5 stimulus mice (**Figure 4C**), while a strong preference for the location of the new stimulus mouse was observed in the recognition test (**Figure 4G**). These data were used to generate binary masks that defined 5 ROIs for subsequent quantification (**Figures 4D–F,H**). Quantification identified a highly significant preference for the new stimulus mouse during the recognition test (**Figures 4I–K**; **Table 2**). Importantly, direct comparison of the data obtained using our new data-driven analysis with those obtained for the same mice using the conventional zone-based analysis (compare **Figures 2C** and **4K**) revealed a substantial improvement both in the statistical significance (one-way ANOVA, *p* < 0.0001 for data-driven analysis vs. *p* < 0.005 for zone-based analysis) and in the magnitude of the SRI (19.0 ± 3.1 sec for data-driven analysis vs. 12.3 ± 9.6 sec for zone-based analysis). These findings confirm that our new data-driven analysis method markedly improves the sensitivity of our paradigm.

We also generated temporal heat maps that depict the distance of all animals from the new stimulus mouse for the entire recognition test duration, using the BALB/c data set as an example (**Figures 5A–F**). This analysis revealed that male BALB/c mice changed their position more often than females (i.e., the color succession changes more rapidly), which was also reflected in a higher average speed of locomotion (recognition test: male = 2.60 ± 0.16 mm/sec; female = 1.41 ± 0.22; *p* = 0.004). Male BALB/c mice also spent more time in close proximity to the new stimulus mouse during the recognition test, particularly in the first minute of the test (**Figure 5D**, increase in green color in males, quantified in **Figures 5G,H**), indicating that they are capable of very rapidly distinguishing between new and familiar stimulus mice.

The observed gender differences are interesting and certainly worth pursuing. The reduced SRI in females may indicate either that female mice are less able to distinguish between 5 mice under the current conditions, or – more likely – that they do not show their recognition through increased exploration of the new stimulus mouse. Considering the higher locomotor activity of males during social exploration, this discrepant behavior may result from inherent differences in gender-specific territorial tasks (Miczek et al., 2001), rather than reflecting a lower social recognition capacity in females. It is also conceivable that the stage of the estrous cycle in female mice played a role, or that sexual maturation and in consequence the development of social skills was influenced by shipment of the female mice during puberty (Laroche et al., 2009). Further investigation of this interesting effect may provide important insights into gender differences in social behaviors.

To finally validate our new behavioral paradigm and analysis tools using a mutant mouse line relevant to psychiatric disorders, we tested mice with heterozygous deletion of PSD-95 (PSD-95^+/-^) (Yao et al., 2004). PSD-95 is a major component of the excitatory synaptic scaffold and plays a key role in development, function and plasticity of excitatory synapses (El-Husseini et al., 2000). Alterations in PSD-95 function and the balance of excitatory and inhibitory synaptic transmission (E/I balance) have been associated with psychiatric phenotypes, including social dysfunction (Yizhar et al., 2011; Tsai et al., 2012; de Bartolomeis et al., 2014), and mice with a full deletion of PSD-95 display abnormalities in social behaviors (Feyder et al., 2010). In contrast, no such changes have been reported in PSD-95^+/-^ mice, despite the fact that a partial reduction in protein levels, rather than a complete deletion, may more accurately reflect the disease contribution of PSD-95 (de Bartolomeis et al., 2014). This lack of a reported phenotype may result partly from the fact that current methods are not sufficiently sensitive to detect the subtle behavioral alterations that might be expected in these mice. Using our new SocioBox paradigm, we find that PSD-95^+/-^ mice show noticeably reduced preference for the new stimulus mouse in the spatial heat map analysis of the recognition test (**Figures 6A,B**). Quantification of these data reveals a significant reduction in the SRI using the data-driven analysis method, but not with the zone-based analysis (**Figures 6C,D**). These data confirm that our SocioBox paradigm is capable of identifying social recognition abnormalities in mutant mouse models, and that the improved sensitivity obtained from the data-driven analysis method can be essential for uncovering subtle disease-related changes. It is also interesting to note that the SRI obtained for the WT mice from the PSD-95 experiment (PSD-95^+/+^ on a C57BL/6J background) is remarkably similar to that seen in the standard inbred C57BL/6J mice from the first experiment (SRI = 14.2 ± 4.7 vs. 12.3 ± 3.9, respectively, for the zone-based analysis; 25.9 ± 6.5 vs. 19.0 ± 3.1, respectively, for the data-driven analysis), despite substantial differences in breeding (bred in-house vs. purchased from Janvier) and housing (17 mice per cage vs. 5 mice per cage). This observation confirms that our SocioBox paradigm can reproducibly measure social recognition across a wide range of experimental conditions.

We conclude that, using a combination of a novel circular testing apparatus, an easy-to-apply experimental paradigm and an advanced set of analysis tools, we have developed a robust and sensitive new behavioral assay to study social recognition in male mice. To our knowledge, this is first report of a paradigm that can assess the ability of mice to discriminate between more than 2 mice using a standardized testing apparatus. Current assays, including the commonly used 3 chambered social approach task (Moy et al., 2004; Kas et al., 2014), are limited in usefulness for studying complex social recognition, since they assess only a binary discrimination between 1 familiar and 1 unfamiliar mouse. Our SocioBox paradigm overcomes this limitation by requiring experimental mice to distinguish an unfamiliar stimulus mouse from 4 newly acquainted stimulus mice, therefore placing a substantially higher social memory load on the mice which renders the assay more sensitive to subtle disease-related changes. Other studies have attempted to address this issue by developing automated systems to analyze patterns of social dynamics in the home cage or in semi-naturalistic environments (Shemesh et al., 2013; Weissbrod et al., 2013; Hong et al., 2015), but these generally require highly specialized and expensive technical equipment that is not widely available. In contrast, the SocioBox paradigm can easily be reconstructed in any standard behavioral laboratory, and the data-driven analysis is based on the open source software FIJI. With this unique combination of sensitivity and ease of implementation, the SocioBox is perfectly poised to assess complex social recognition in mouse models of psychiatric disorders. In particular, this assay will be valuable for studying models of autism and schizophrenia, both of which involve core deficits in social skills, including specific impairments in social and face recognition (Boucher and Lewis, 1992; Calkins et al., 2005; Tanaka et al., 2010; Meyer-Lindenberg and Tost, 2012). As such, the SocioBox represents an important expansion to the current repertoire of tests available to assess social skills in mice, and in combination with other behavioral paradigms measuring complementary aspects such as sociability, it will greatly facilitate the comprehensive analysis of social dysfunction in mouse models of psychiatric disorders.

## Author Contributions

HE conceived the study; HE, DK-B, and DW designed the study; DW performed experiments on all WT cohorts, supported by AR and DK-B; FD performed PSD-95^+/-^ experiment; MM developed visualization and analysis tools; HE, DW, DK-B, MM, and ED analyzed experiments; OMS provided the PSD-95 knockout mouse line and gave conceptual advice; DK-B, DW, MM, and HE wrote the paper. All authors discussed the results and commented on the manuscript at all stages.

## Conflict of Interest Statement

The authors declare that the research was conducted in the absence of any commercial or financial relationships that could be construed as a potential conflict of interest.
