# Supplementary material for: The SocioBox: A Novel Paradigm to Assess Complex Social Recognition in Male Mice
Source: Front Behav Neurosci. 2016 Aug 11;10:151. doi: 10.3389/fnbeh.2016.00151 (PMC4980394; doi:10.3389/fnbeh.2016.00151)

The SocioBox: A novel paradigm to assess complex social recognition in male mice

Dilja Krueger-Burg[1,§], Daniela Winkler[1,2,§], Mišo Mitkovski[1,§], Fernanda Daher[1],
Anja Ronnenberg[1], Oliver M. Schlüter[2,3], Ekrem Dere[1,2], and Hannelore Ehrenreich[1,2*]

SUPPLEMENT: DEVELOPMENT OF THE SOCIOBOX PARADIGM

In the course of the development of the SocioBox paradigm, we tested multiple versions of the chamber and experimental paradigm. Since the insights gained from these preliminary experiments may be valuable to others, we have briefly summarized the key points below.

Rectangular chamber version 1: The initial design consisted of a rectangular chamber in which five inserts were aligned along a straight corridor. Social interaction was recorded as time spent in each of five interaction zones immediately in front of the inserts.

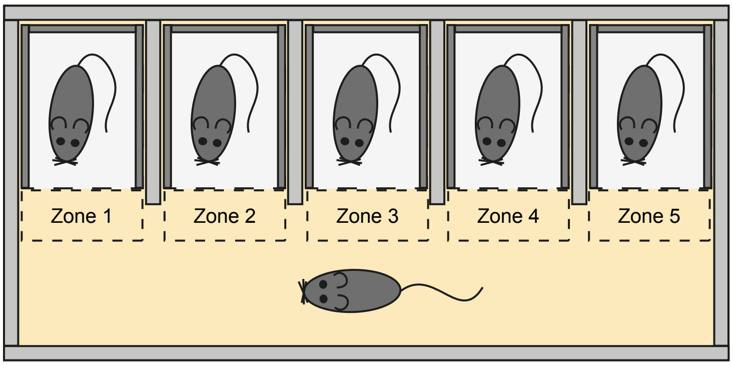

Two issues became obvious with this design in preliminary tests: (1) The experimental mice spent substantial amounts of time exploring or sitting in the corners of the chamber, and they frequently entered the interaction zones in front of the outer stimulus mice (zones 1&5) without actually interacting with the stimulus mice. This artificially increased the automated recording of the interaction time with the outer stimulus mice. (2) In this design of the chamber, which lacked a movable partition to separate the experimental mouse from the stimulus mice (see below), the experimental mice spent the first 5min of the interaction phase exploring the chamber or grooming to recover from handling, which confounded our measurements. These issues were addressed in a second version of the rectangular chamber as described below.

Rectangular chamber version 2: The second version of the rectangular chamber included empty wings on the side of the box to allow for greater spatial separation of the corners from the interaction zone in front of the outer stimulus mice (issue 1 above). In addition, we included a moveable partition that allowed us to separate the experimental mouse from the stimulus mice for the first 5min of the test (issue 2 above).

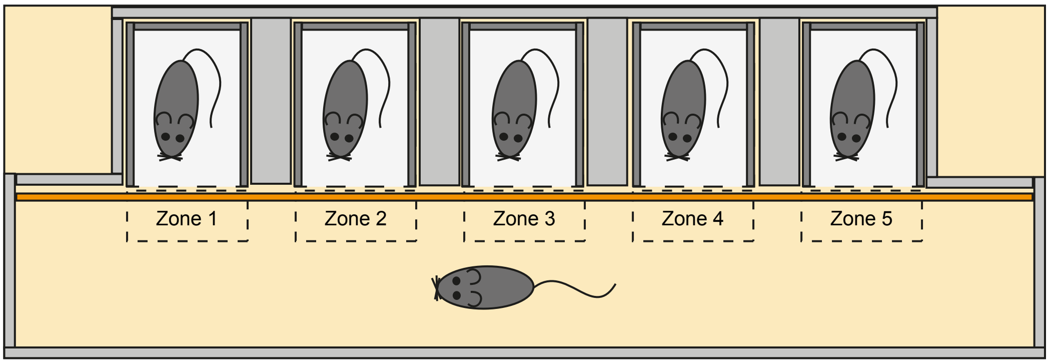

To test this apparatus, we conducted an experiment using male C57BL/6N mice (n=10) as experimental mice and male C3H mice as stimulus mice. All mice were habituated to the apparatus for 3 consecutive days as described in the online methods for the final SocioBox chamber. They were then subjected to a test consisting of 3 phases, with the same handling and cleaning conditions as described for the SocioBox chamber (see [a] below). The position of the 5 stimulus mice was randomized across experimental mice (see [b] below), and the data were later aligned to the position of the original/new stimulus mouse.

a. Phases of the test session

	Duration interval stage	Duration interaction stage	Time in homecage between phases
Exposure 1	5 min	10 min	24 h
Exposure 2	5 min	10 min	5 min
Recognition test	5 min	10 min	End of test

b. Scheme for placement of the stimulus mouse

	Zone 1	Zone 2	Zone 3	Zone 4	Zone 5
Test mouse 1	S[1]	S[2]	S[ori]/ S[new]	S[3]	S[4]
Test mouse 2	S[4]	S[1]	S[2]	S[ori]/ S[new]	S[3]
Test mouse 3	S[3]	S[4]	S[1]	S[2]	S[ori]/ S[new]
Test mouse 4	S[ori]/ S[new]	S[3]	S[4]	S[1]	S[2]
Test mouse 5	S[2]	S[ori]/ S[new]	S[3]	S[4]	S[1]

Under these conditions, the experimental mice showed significant exploration of the stimulus mice but very little recognition of the new stimulus mouse during the social recognition test.

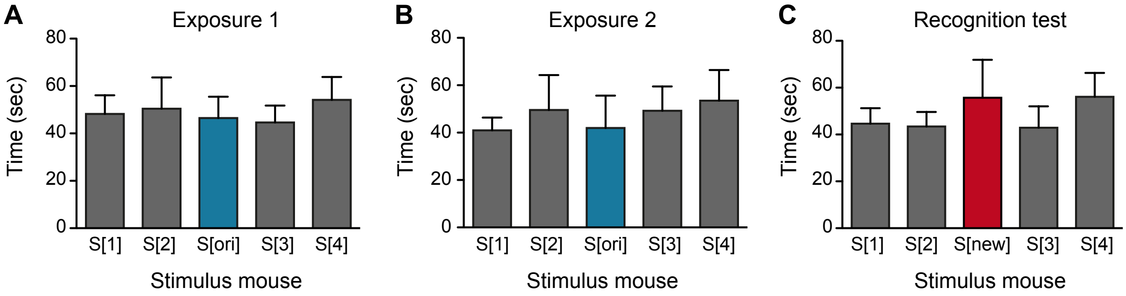

To investigate whether spatial biases in the exploration by the experimental mice may contribute to the lack of a recognition effect, we analyzed the data from exposure 1 and exposure 2 by interaction zones without aligning to the original stimulus mouse (since in exposures 1 and 2, there is no conceptual difference between the original stimulus mouse and the constant stimulus mice from the vantage point of the experimental mouse). This analysis revealed a significant spatial bias for investigation of the outer stimulus mice (zone 1 and zone 5), which is likely to substantially contribute to the variability in the data obtained for the social recognition test. To overcome this issue, we developed the circular SocioBox version as described in the main paper.

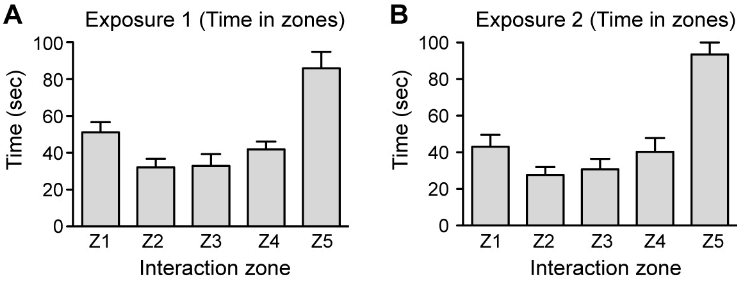

Supplement: Supplementary file 1 [file Data_Sheet_1.PDF]
